# India's family planning market and opportunities for the private sector: An analysis using the total market approach

**DOI:** 10.1002/hpm.2753

**Published:** 2019-03-15

**Authors:** Arupendra Mozumdar, Rajib Acharya, Subrato Kumar Mondal, Amit Arun Shah, Niranjan Saggurti

**Affiliations:** ^1^ Reproductive Health Program Population Council New Delhi India; ^2^ Health Office United States Agency for International Development New Delhi India

**Keywords:** family planning, India, National Family Health Survey, private sector, total market analysis

## Abstract

The private (commercial) sector in India can complement public sector for family planning services, but the roadmap to engage these two sectors remains a challenge. The total market approach (TMA) offers a strategy by understanding the comparative advantage of public, commercial, and nonprofit sectors. We estimated TMA indicators using data of four rounds of the National Family Health Surveys: 1992‐93, 1998‐99, 2005‐06, and 2015‐16. The contraceptive prevalence of modern methods in India did not increase in recent years, but the number of users increased, and so did the market size for the commercial sector. In rural areas, the current market size in 2015‐16 (75 million) failed to reach its potential size in 1992‐93 (84 million). In urban areas, the market of modern contraceptives is mostly composed of the users from higher wealth, and a high percentage of users obtain contraceptives from subsidized sources. The family planning market of northern part of Bihar and Uttar Pradesh and of Northeast India are in the “early” stage and need more demand generation; “matured” markets are mostly concentrated in and around big metros. Subsidization in urban areas should be offered to the targeted population who need family planning products and services at low cost.

## 1 INTRODUCTION

At the London Summit, Family Planning 2020 (FP2020) partnership of governments, donors, and civil society organizations committed to provide quality family planning services to an additional 120 million women across the world. In low‐ and middle‐income countries, where the resources for health care are limited, the most effective strategy to meet the demand of family planning products and services requires continued engagement and coordinated approach from all sectors of health care: the public sector, the private nonprofit sector, and the private commercial sector.^1^ The use of family planning products and services may increase if these three sectors can work in tandem with their competitive advantage among different segments of the population, such as public and nonprofit sectors to provide subsidized services for needy consumers while maintaining a sustainable commercial provision for consumers who can afford to pay. This approach is often referred as the “total market approach” (TMA), which aims for the growth of the family planning market by emphasizing the efficient use of all available public, nonprofit, and private commercial sector resources and infrastructure, to improve access for all segments of the population and to provide sustainability of the market.^2^

Using this approach, this article evaluates the potential role of private sector in the family planning market of India, which is traditionally dominated by the public sector providing family planning products to couples free of cost. Understanding the comparative roles and advantages of each of these sectors will likely help program managers to frame effective plans for engaging private sector to increase use of family planning products and services.

### 1.1 The global context

The Market Development Approaches Working Group of the Reproductive Health Supplies Coalition strongly advocates to reorient government health policymakers and reproductive health program managers from a single sector focus to adopting a total market perspective and thus increase access to reproductive health products and services efficiently and equitably.^1^ In the last decade, an increasing number of documents and reports on TMA had come out to inform policymakers and program managers about the development of the market of family planning products and services through different TMA indicators.^1^, ^3^, ^4^, ^5^ A compendium of TMA indicators was documented under the Evidence project by Measure Evaluation, which recommended 26 key TMA indicators grouped under 11 topics with four broad categories: market size, market accessibility, market sustainability, and market equity.^2^

Meekers et al^2^ further recommended that TMA planning should aim to track and analyze as many of these indicators as feasible, and priority should be given to those indicators that can be measured with existing standardized surveys to minimize the measurement burden. Demographic and Health Surveys (DHSs) provide comparable data on key TMA indicators from a nationally representative sample of women to inform policies and programs in family planning and were periodically conducted over 90 countries. The estimation of key TMA indicators has been done for the countries like Paraguay,^6^ Cambodia,^7^ Myanmar,^4^ Caribbean countries,^8^ Bangladesh,^9^, ^10^ Indonesia, Mexico and Egypt,^11^ Honduras and Senegal,^12^ Romania, Thailand, and Turkey,^13^ Uganda, Botswana, Lesotho, Mali, South Africa, and Swaziland.^14^

### 1.2 The Indian context

India is the most populous country among low‐ and middle‐income countries and committed to provide quality family planning services to 48 million additional women. However, the roadmap to achieve this goal remains a challenge, majorly due to the already overburdened public sector.^15^ The TMA offers a strategy to understand this challenge accounting for available resources of public, commercial, and nonprofit sectors; however, so far, only a few studies have attempted to understand India's family planning market,^16^, ^17^ but none has examined the same using globally accepted TMA indicators.

The National Family Health Survey (NFHS), which is the DHS for India, provides data for estimation of many TMA indicators over a time span from 1992‐93 to 2015‐16 through four rounds of the survey.^18^, ^19^, ^20^, ^21^ NFHS uses similar questionnaire and follow a standardized procedure of data collection and thus making data from different rounds comparable. Further, the large sample size facilitates the analysis of TMA indicators for different segments of the population.

This article attempts to estimate contributions of different sectors to the family planning market in India and their changes over the last two decades. Further, the total market indicators for India's family planning market and their changes have been calculated using NFHS data. The article also attempts to locate geographies where the private sector has the potential for sustainable growth within the family planning market in India.

## 2 MATERIALS AND METHODS

### 2.1 Data source

We used women's data from four rounds of the NFHS: 1992‐93, 1998‐99, 2005‐06, and 2015‐16. The NFHSs are designed to provide data on family planning comparable across the rounds, despite need‐based changes were made in each round with changing priorities of the country's family planning program. Data from the following family planning indicators were used in the analysis.

#### 2.1.1 Current use of family planning methods

Women who reported using a method to delay or avoid pregnancy were asked to specify the method they were using at the time of the survey. This information was used to calculate the contraceptive prevalence rate and the current method mix. For this article, a woman and/or her partner were considered as users of modern contraceptives if they were using any of the following methods of family planning—female sterilization, male sterilization, intrauterine contraceptive device (IUCD), injectables, implants, pill, condom, female condom, diaphragm, foam or jelly, or other modern methods.

#### 2.1.2 Unmet need for family planning

All four rounds of NFHS asked several questions to the women respondents that enable us to calculate the percentages of women who had met or unmet need for family planning at the time of the survey. For this article, for all four rounds of the survey, we use the 2012 definition of unmet need by Bradley and colleagues.^22^ The unmet need for family planning has been used to calculate the total demand for family planning, which is the proportion of currently married women (15‐49 y) who are either using any contraceptive method or having an unmet need for family planning.

#### 2.1.3 Sources of the most recent method of family planning

In all rounds of NFHS, current users of modern family planning methods were asked from where they obtained the method they were using for the last time before the survey. Possible responses included the public health sector sources (eg, government hospital, government health center, family planning clinic, mobile clinic, fieldworker, or other public sector facility/health worker), the private health sector sources (eg, private hospital/clinic, pharmacy, private doctor, mobile clinic, fieldworker, or other private medical sector facility/health worker), the nonprofit health sector sources (eg, nongovernment organization or trust hospital/clinics), and other sources (eg, shops, friends/relatives, or others). If the women were unable to report if the source of family planning service/product was public or private in nature, they reported the name of the place, which was later recoded into the appropriate response code.^23^

The information about the source of family planning method has been used as a proxy for the market share of the public, private (commercial), and nonprofit sectors. However, the answer codes are not detailed enough to accurately distinguish between commercial sector, nonprofit making sector, and social‐marketing sector.

#### 2.1.4 Wealth index

We used the standard wealth index provided in the datasets. The DHS Wealth Index is a composite indicator based on a combination of household ownership of a series of assets and access to various amenities and services. Principal component analysis was used to assign weights to the household assets and amenities, and a wealth score for each household was generated.^24^, ^25^, ^26^ Households were then ranked based on the wealth score and subsequently classified into quintiles, where the 20% of the lowest scores comprise the first quintile and grouped as “poorest.” The subsequent quintiles were grouped as “poorer,” “middle,” “richer,” and “richest.” The wealth index of the respondent's household was given with the dataset. The recoded and revised datasets of the NFHS 1992‐93 and NFHS 1998‐99 also provide the wealth index score of those two rounds along with survey data, which was utilized in this article.

### 2.2 Total market approach indicators

The Market Development Approaches Working Group of the Reproductive Health Supplies Coalition^1^ recommended four broad characteristics of the market that should be tracked to monitor the growth and maturity of the market. Those are market size, market equity, market accessibility, and market sustainability.

#### 2.2.1 Market size

We defined the size of the family planning market by the number of married women of reproductive age, who were using any modern contraceptive method. We used sampling weights provided in the dataset and the total number of women aged 15 to 49 years in the country for the survey year to estimate this number.^27^ Data on the total number of women were obtained from the United Nations' database of World Population Prospects for the survey years. The base populations of the years 1990, 1995, 2005, and 2015 were used for the survey years 1992‐93, 1998‐99, 2005‐06, and 2015‐16, respectively.

To assess the total potential demand for the family planning services or products, the number of women with unmet need was also calculated. Adding the total number of women with unmet need with the number of current users indicates the potential of the market growth in terms of numbers of potential customers of family planning services and products.

#### 2.2.2 Market equity

In this article, market equity was studied by estimating the percentage of users who obtained their last method from a subsidized or unsubsidized source disaggregated by wealth quintile and place of residence (urban/rural areas). We also calculated the change in the composition of the wealth index among users of modern contraceptive methods for both subsidized and unsubsidized sources, as a proxy indicator for the change in the number of customers of family planning across different socioeconomic classes.

We examined whether the market of specific contraceptive is dominated by one brand or player or not, by estimating the market share of subsidized and unsubsidized brands available on the market. The information on types of the brands for pills and condoms was not available for NFHS 2015‐16, so it was calculated from the NFHS 2005‐06 data. Because women were less likely to report the type of brand of condom than men and men were less likely to report correctly about pill, the use of type of brands for pills and condoms were estimated from couples' data—for pills, we used women's data, and for condoms, we used husbands' data.

#### 2.2.3 Market accessibility

The market accessibility indicators were used to assess the extent to which potential users have access to family planning products and services. Given that no direct data are available to measure geographical and financial access for women to use contraception, in this article, we used two proxy measures: percentages of married women with unmet need who reported that they were not using any family planning method because of “lack of access” to measure geographical barrier, or because of the contraceptives “cost too high,” to measure financial barrier.

#### 2.2.4 Market sustainability

The sustainability of family planning markets in India, that is, its potential to be a self‐sustaining market, should ideally be assessed through three indicators—the total market value, the market share held by market leaders, and market subsidies. However, NFHS does not provide data for the first two indicators, and hence, in this analysis, we assessed market sustainability by the level of family planning use and subsidization.

Total market approach aims to strengthen the sustainability of the market through increased involvement of commercial or unsubsidized sector, targeting the free and subsidized services and products only for those who cannot afford to pay. The family planning market in India remains dominated by government subsidies with more than 75% of the use of modern contraception being female sterilization a free service largely provided through public health facilities. A market dominated by subsidized brands and services has the potential to discourage market growth and sustainability by inhibiting the participation of the commercial sector. Therefore, to create inroads for the commercial sector and developing future intervention strategies, it is necessary to assess how developed a market is for family planning services and products. The stage of market development is determined by the size of the market and the provision of unsubsidized products and services for the commercial sector (Barnes et al. 2012).

Using the data from NFHS 2015‐16 and following the classification of the total market initiative proposed by the “Reproductive Health Primer,”^1^ each of India's 640 districts were categorized into one of the three stages of contraceptive market development: early, developing, or mature. We categorized a district as at the “early” stage of market development when the use of modern contraceptives (mCPR) is less than 25%. In contrast, a district is categorized as at “mature” stage when mCPR is more than 55% with less than 50% share of subsidized sources. All other districts are categorized as at “developing” stage. The details of classification of districts by their stage of development of market are given in Box Box 1 Classification of districts by stages of market development of family planning services and products.

**Box 1 Classification of districts by stages of market development of family planning services and products**

**Table:** 

Stages of FP Market Development	Number of Districts	Percentage (N = 640)
Early (mCPR less than 25%)	90	14.1
Developing (mCPR between 25‐55% or more than 50% share of subsidized sources)	541	84.5
Mature (mCPR more than 55% with less than 50% share of subsidized sources)	9	1.4

In order to understand the variation in the family planning market for different population segments, stages of development of family planning market for each district was calculated for segments with contrasting characteristics, such as among women who are living in urban vs rural areas, women with no education vs women with higher secondary and above education, and between women from families in the “poorest” wealth quintile vs the “richest.” We used state wealth index scores instead of the national wealth index, in the district level analysis of the stages of market development between two extreme wealth quintiles, to get enough sample for two extreme wealth quintile groups in each district.

#### 2.2.5 Ethical consideration

The analysis in this article was done using secondary data of four rounds of NFHS available in the public domain after removing the individual identification. The ethical clearance for each round of NFHS was obtained from Institutional Review Boards (IRB) of the International Institute of Population Science, Mumbai, India, and the Technical Assistance Unit of the respective survey round. Each respondent of the survey gave voluntary consent to participate in the survey. The details of the ethical consideration of NFHS can be found in full reports of all four rounds of NFHS.

## 3 RESULTS

**Figure 1:**
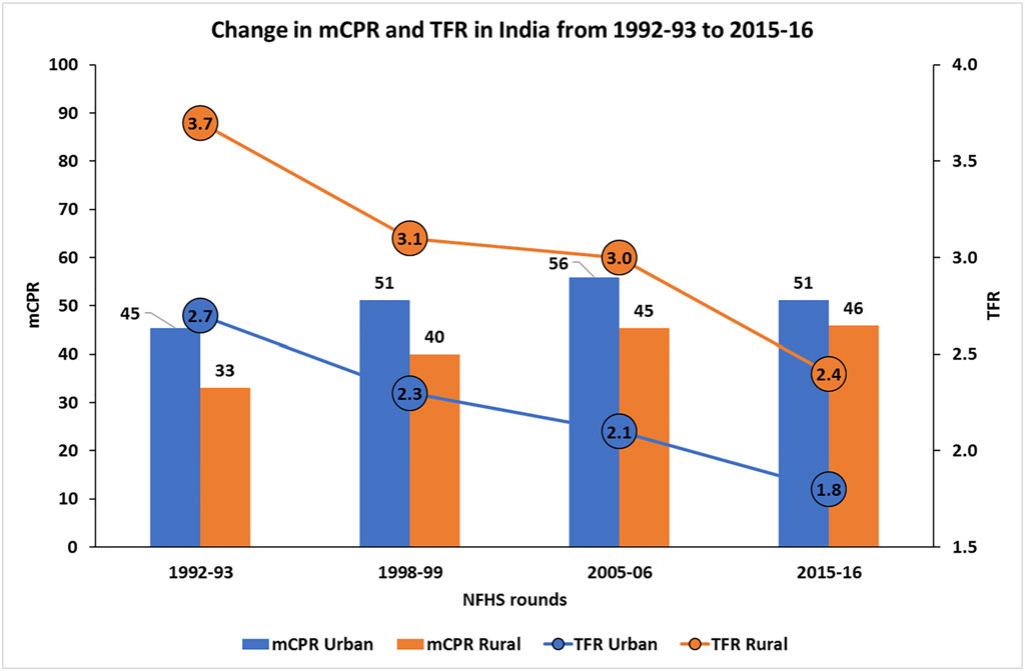
Changes in modern contraceptives prevalence rate (mCPR) among currently married women of reproductive age and total fertility rates (TFR) in urban and rural areas, India 1992‐93 to 2015‐16 [Colour figure can be viewed at http://wileyonlinelibrary.com]

We first examined the trends in fertility and family planning in India during the last two decades. Data from four rounds of NFHS show that the total fertility rate (TFR) in India declined sharply in both urban and rural areas during the period 1992‐93 to 2015‐16—from 3.7 in 1992‐93 to 2.4 in 2015‐16 in rural areas and from 2.7 in 1992‐93 to 1.8 in 2015‐16 in urban areas. In the last decade, the urban‐rural gap in TFR reduced from 0.9 to 0.6 (Figure 1).

In contrast, and unexpectedly, the overall mCPR did not change much in the last 10 years in India; in fact, mCPR declined from 56% in 2005‐06 to 51% in 2015‐16 among women living in urban areas. Only a 1% increase in mCPR was recorded for rural areas from 2005‐06 to 2015‐16.

### 3.1 Sources of contraceptive methods

**Figure 2:**
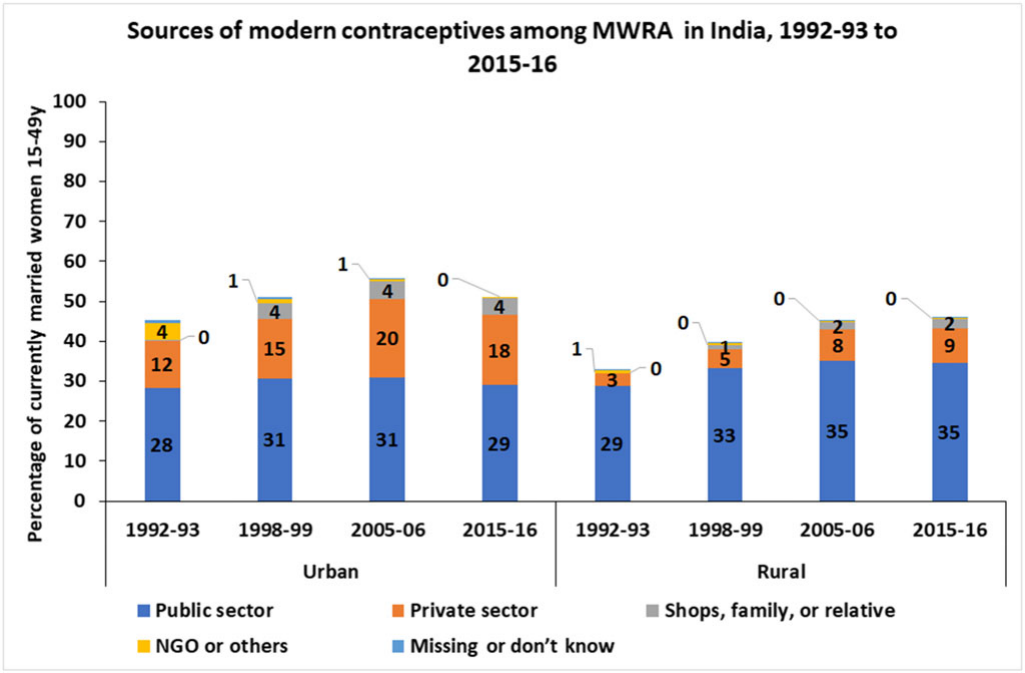
Sources of modern contraceptive method among currently married women 15‐49 y from urban and rural areas, India 1992‐93 to 2015‐16 [Colour figure can be viewed at http://wileyonlinelibrary.com]

Nearly 30% of currently married women (15‐49 y) obtained the most recent contraceptive method from the public sector, both in urban and rural areas (Figure 2). The percentage for the public sector has increased from 29% to 35% in rural areas from 1992‐93 to 2015‐16 but did not change in the urban areas (28% to 29%). The percentage of married women who obtained their most recent supply of contraceptive from a source in the private health sector has increased both in urban and rural areas. In the urban areas, it has increased from 12% in 1992‐93 to 20% in 2005‐06 before dipping to 18% in 2015‐16. In the rural areas, the percentage of women who obtained modern contraceptives from the private health sector increased steadily from 3% in 1992‐93 to 9% in 2015‐16.

### 3.2 Change in market size

**Figure 3:**
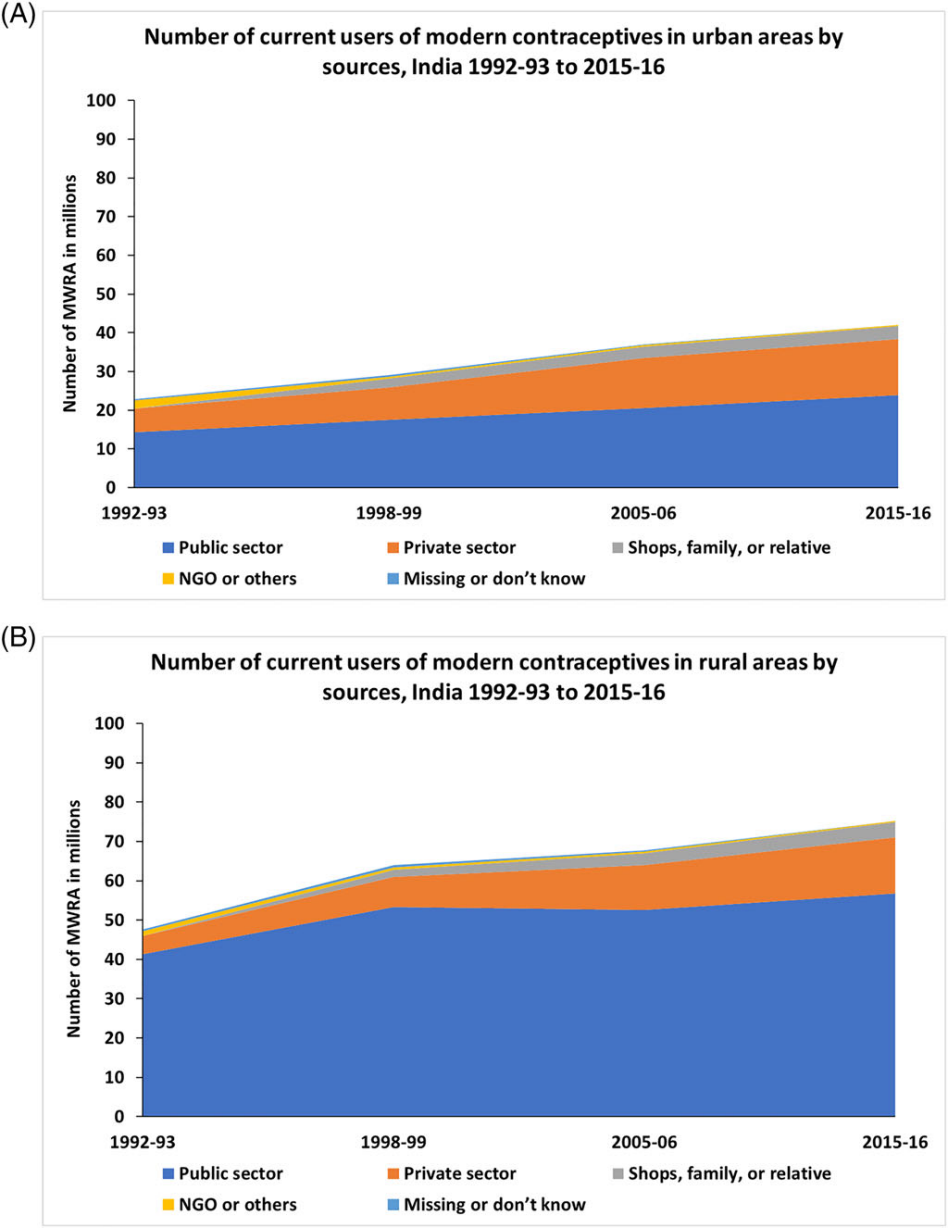
Number of users of modern contraceptives among currently married women 15‐49 y, India 1992‐93 to 2015‐16 [Colour figure can be viewed at http://wileyonlinelibrary.com]

Although the mCPR for India decreased in the last 10 years, the market size for modern contraceptives—ie, the number of users of modern contraceptives among currently married women (15‐49 y)—has increased from 2005‐06 to 2015‐16, and much of this increase was the contribution of the private sector (Figure 3). The market size of the modern contraceptives, ie, the number of users of modern contraceptives among currently married women (15‐49 y) has increased from 70 million in 1992‐93 to 117 million in 2015‐16. In urban areas, the number of users of modern contraceptive who obtained the method from the private health sector in 1992‐93 was about 6 million, which increased to 14.5 million in 2015‐16. In rural areas, the number of users of modern contraceptives from the private health sector increased from 4.5 million in 1992‐93 to 14 million in 2015‐16.

**Figure 4:**
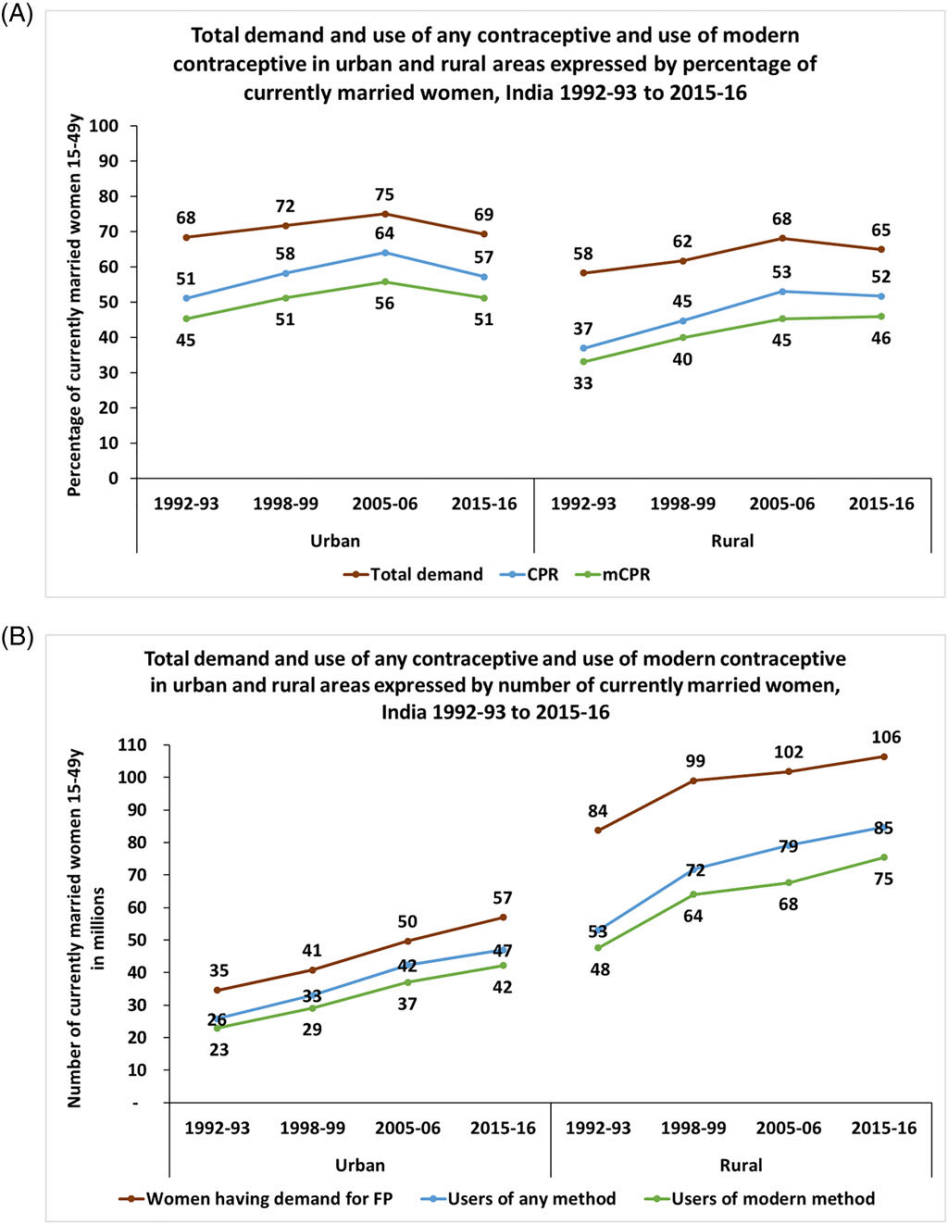
Total demand, use of any contraceptive, and use of modern contraceptive among currently married women of 15‐49 y (CMW) from urban and rural areas expressed by percentage and number of CMW, India 1992‐93 to 2015‐16 [Colour figure can be viewed at http://wileyonlinelibrary.com]

The total demand for family planning in India increased from 68% in 1992‐93 to 75% in 2005‐06 but declined to 69% in 2015‐16 (Figure 4A). In rural areas, the total demand experienced a 10% point increase between 1992‐93 and 2005‐06, growing from 58% to 68%, but slightly dropped to 65% in 2015‐16.

The number of women, who has demand for family planning, consistently increased in both urban and rural areas (Figure 4B) between 1992‐93 and 2015‐16 by about 22 million each. A gap of 12 million existed in 1992‐93 between the number of women in demand for family planning and the number of women who are using any modern family planning method. The gap increased to 15 million for urban areas in 2015‐16. The gap between demand and usage of family planning method was much wider in rural areas than in urban areas; however, the gap reduced from 36 million in 1992‐93 to 31 million in 2015‐16.

### 3.3 Change in market share of subsidized and unsubsidized family planning services/products

Table 1 provides data on percent of women who were using each of the four selected modern contraceptive methods during each round of the survey and proportion of users of these methods who sourced them from unsubsidized sectors. In 1992‐93, 36% of married women of reproductive age were using a modern contraceptive method, but only 20% of them sourced these methods from the unsubsidized sector, ie, did not obtain the method from any public health sector source or the nonprofit sector source. Share of the unsubsidized sector in the source of modern methods has increased from 20% in 1992‐93 to 31% in 2015‐16, concurrent with an increase in modern method use in the same period. In India, the method mix is highly skewed for female sterilization, and it remained above 75% of the total usage of modern methods; the share of unsubsidized sector remained low too—only about 18% of the female sterilizations were done in the unsubsidized sector in 2015‐16. Further, among those few (only 2%) who were using an IUCD, about 40% obtained it from private health clinics, and it remained somewhat consistent over the period from 1992‐93 to 2015‐16. Pills and condoms have been two main nonclinical methods of family planning used by couples in India, and about 68% to 84% of the users of these two methods obtained them from unsubsidized health sectors such as private health clinics, or shops. During the same period, increase in the use of these two methods has been modest—1% to 4% for pills and 2% to 6% for condoms.

**Table 1:** Use of selected contraceptive method among currently married women 15 to 49 years and proportion of users obtained the method from unsubsidized sources, India 1992‐93 to 2015‐16

Contraceptive Method	NFHS 1992‐93	% Unsub. in 1992‐93	NFHS 1998‐99	% Unsub. in 1998‐99	NFHS 2005‐06	% Unsub. in 2005‐06	NFHS 2015‐16	% Unsub. in 2015‐16
Modern method	36.3	19.9	42.8	21.6	48.5	29.0	47.8	30.6
Female sterilization	27.3	13.2	34.1	13.1	37.3	15.8	36.0	17.5
IUCD	1.9	35.6	1.6	42.9	1.7	52.0	1.5	40.4
Pill	1.2	68.4	2.1	75.0	3.1	83.8	4.1	72.2
Condom	2.4	81.2	3.1	76.3	5.2	85.7	5.6	82.5

Abbreviation: % Unsub. = percentage of women obtained the method from an unsubsidized source.

The distribution of couples in 2005‐06, who used pills and condoms, showed that social marketing brands were the most popular brands among users of these methods (Table 2). It is noteworthy that even among those users who obtained these methods from unsubsidized sources, 43% to 59% were using social marketing brands. Similarly, in the case of men using condoms, about 42% to 60% of those who obtained condom from the unsubsidized sector used social marketing brands. The use of commercial brands of pills and condoms was higher in urban areas among those women and men who obtained these two methods from unsubsidized sources.

**Table 2:** Percentage distribution of couples who were using pills and condoms by brand type and source in urban and rural areas, India 2005‐06

	Wife Using Pill		Husband Using Condom	
	Subsidized Source	Unsubsidized Source	Subsidized Source	Unsubsidized Source
Urban				
Brand type				
Social marketing brand	81.4	58.5	61.9	42.1
Commercial brand	14.0	21.1	10.2	44.9
Other	0.0	13.8	1.7	2.6
Don't know	4.7	6.6	26.3	10.4
Rural				
Brand type				
Social marketing brand	73.0	42.8	71.0	59.7
Commercial brand	3.2	14.8	2.6	18.9
Other	7.1	36.2	0.0	1.6
Don't know	16.7	6.2	26.4	19.8

### 3.4 Changes in market equity

**Figure 5:**
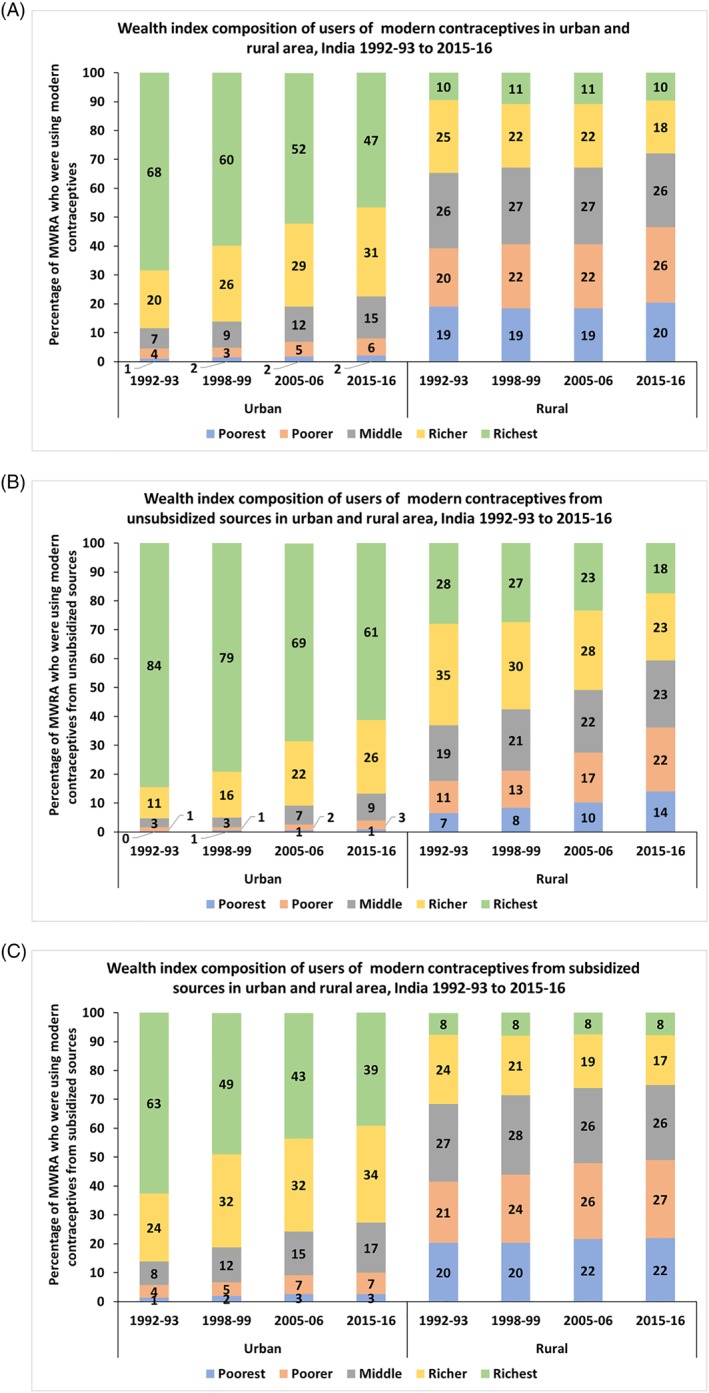
Wealth index composition of users of modern contraceptive who obtained the method from unsubsidized and subsidized sources living in urban and rural areas, India 1992‐93 to 2015‐16 [Colour figure can be viewed at http://wileyonlinelibrary.com]

The mCPR in India increased across the wealth quintile over the last 10 years, except for the richest wealth quintile.^20^, ^21^ However, the wealth index composition of the users of modern contraceptives shows that in the urban areas, nearly 50% (2015‐16) of the users were from the highest wealth quintile, a steady decline from 70% in 1992‐93 (Figure 5A). The wealth quintile composition of the modern contraceptive users in the rural areas was uniform and remained unchanged over the last two decades.

In the rural areas, among those who obtained the method from the unsubsidized sector, the percentage of users from poorest or poorer wealth quintile doubled from 18% in 1992‐93 to 36% in 2015‐16, and correspondingly, the share of two wealthiest groups declined from 63% to 41% (Figure 5B). In urban areas, among those who obtained the method from the unsubsidized sector, the distributions were more uneven, and the share of the wealthiest group alone was 84% in 1992‐93, which has sharply declined to 61% in 2015‐16.

The composition of wealth quintile remained almost unchanged among the users of modern methods from subsidized sources in rural areas. However, among the users in urban areas, who received modern contraceptives from the subsidized sector, 39% were from the richest wealth quintile in 2015‐16, a decline from 63% in 1992‐93. In urban areas, a very low percentage of users, who obtained the method from subsidized sources, were from “poorer” or “poorest” wealth quintile (Figure 5C).

### 3.5 Changes in market accessibility

Percentage of nonusers who reported “lack of access” as a reason for not using any family planning method was less than 1% for most of the wealth groups (Table 3), and these percentages did not change across the rounds of the survey.

**Table 3:** Percentage of married women with unmet need for family planning reported the cause of not using any family planning method as “lack of access”, separated by urban/rural area and by wealth index, India 1992‐93 to 2015‐16

Wealth Index	1992‐93		1998‐99		2005‐06		2015‐16	
	Urban	Rural	Urban	Rural	Urban	Rural	Urban	Rural
Poorest	‐	0.5	‐	1.1	<0.1	1.2	<0.1	0.4
Poorer	1.6	0.2	0.7	1.4	‐	1.3	0.3	0.5
Middle	1.4	0.1	‐	0.4	0.5	0.5	1.2	0.8
Richer	0.2	‐	0.2	0.2	1.4	0.8	0.9	0.9
Richest	0.1	0.6	0.1	0.2	0.3	<0.1	0.4	0.7

Percentage of nonusers, who reported that contraceptives “costs too much” as the main reason for not using any family planning method, increased from 1992‐93 to 2015‐16 for all wealth index, and they were less than 5% for most of the wealth groups (Table 4). In every round, a slightly higher percentage of women with unmet need from the “poorest” and “poor” wealth groups reported the cost of contraceptives as one of the reasons for nonuse.

**Table 4:** Percentage of married women with unmet need for family planning reported the cause of not using any family planning method because contraceptive “costs too much,” separated by urban/rural area and by wealth index, India 1992‐93 to 2015‐16

Wealth Index	1992‐93		1998‐99		2005‐06		2015‐16	
	Urban	Rural	Urban	Rural	Urban	Rural	Urban	Rural
Poorest	2.6	1.5	5.1	2.9	6.2	7.3	9.2	4.8
Poorer	<0.1	0.8	2.8	2.4	7.2	5.2	4.9	4.2
Middle	0.9	0.4	0.3	0.9	3.8	2.0	3.8	2.8
Richer	0.5	0.5	0.7	0.5	2.3	0.6	3.1	2.3
Richest	0.2	‐	0.1	0.2	0.4	0.2	1.7	1.1

### 3.6 Market sustainability: Current stages of market development

**Figure 6:**
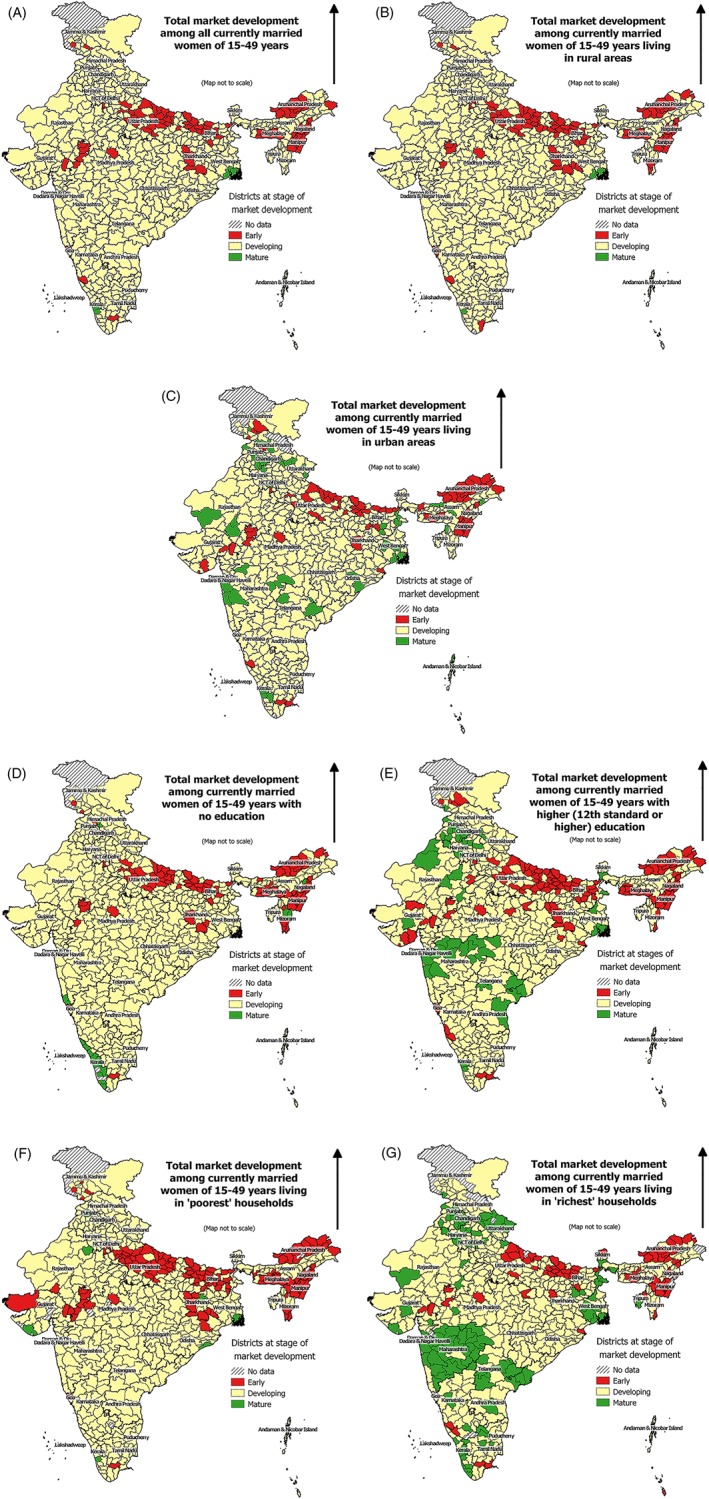
Stages of market development for contraceptive use in India among currently married women of 15‐49 y and by selected socioeconomic groups, 2015‐16 [Colour figure can be viewed at http://wileyonlinelibrary.com]

As described in Section 2 of this article (Box Box 1 Classification of districts by stages of market development of family planning services and products), each of the 640 districts of India was classified into one of the three groups by their stages of development of family planning market: early, developing, and mature. The family planning market in most districts (n = 541, 85%) was at the “developing” stage. The market was at the “early” stage in 90 districts and at the “mature” stage in only 9 districts (Figure 6A).

The market for family planning services and products in urban areas was somewhat more developed than in rural areas (Figure 6B,C). The market in urban areas of 78 districts (12%) was at the “early” stage, 52 districts (8%) at the “mature” stage, 500 districts (79%) at the “developing” stage, compared with 97 (16%), 8 (1%), and 516 (83%) districts, respectively, in the rural areas. The market of family planning services and products among women with higher secondary or higher education showed that in 56 districts, the market was at the “mature” stage, but at the same time, the market was at the “early” stage in 120 districts. Only in 19 districts the family planning market among uneducated women was in the “mature” stage (Figure 6D,E). Among women from poorest households, the family planning market of only seven districts was at the “mature” stage, and in 137 districts, the market was at the “early” stage (Figure 6F). Among the women from “richest” wealth quintile, the market of family planning services and products in 114 districts were in the “mature” stage, but still in 70 districts, the market was at the “early” stage (Figure 6G).

Across the segments of the population, the family planning markets in the districts of the northern part of Bihar and Uttar Pradesh, and in districts from Northeast India, were at the “early” stage. The market was in the “mature” stage in the districts in and around the major metro cities, such as Kolkata, Delhi, and Mumbai. In South India, where the use of modern contraceptives is high, districts were in “developing” stage because of a higher proportion of usage from the public health sectors (subsidized).

## 4 DISCUSSION

In this article, we estimated major TMA indicators for India using the nationally representative data on family planning for the first time and demonstrated the changes in family planning market of the country over the last two decades. The data required to estimate all the TMA indicators are not available in the public domain. However, a number of standardized indicators could be calculated for India using the NFHS data.

We found a 67% increase in the size of India's market of family planning services and products in the last 20 years, especially the urban areas that experienced a 100% increase in users of family planning. But the increase in users is mostly due to an increase in the population of married women of reproductive age rather the increase in contraceptive prevalence rate.

The total demand for family planning signals the potential growth of the market. Although the percentage of women in demand for contraceptives in India did not change much over the last two decades, the number of women who are in demand increased steadily. This increase in the potential market of contraceptives gives space for both public and private sector to grow.

The contribution of the private (commercial) sector in market size of contraceptives increased in the last two decades with the increase of market size, but the increase slowed down in the last 10 years. In the urban areas, where the private sector had the maximum opportunity to grow, the number of contraceptive users from private sector increased by more than 10 million during 1992‐93 to 2005‐06 but by only 2 million in last 10 years (from 2005‐06 to 2015‐16). Since 2005‐06, in the urban areas, the proportion of users of modern methods, who obtained their contraceptives from the private sector, declined by two percentage points. This finding along with the fact that demand for family planning has increased over time indicate the importance of strengthening the private sector supply side in the urban areas. In other words, the gap between the potential market size and current market size decreased in rural areas, but increased in the urban areas, indicating that, in spite of a better scope for market growth, the supply of family planning services and products did not improve in urban areas, compared with that of rural areas. In rural areas, however, the market size in 2015‐16 (75 million) failed to reach its potential size calculated from the 1992‐93 data (84 million) (Figure 4B).

Total market approach refers to having the subsidized services and products for needy consumers while maintaining a sustainable commercial provision for consumers who can pay. A different programmatic approach can, however, achieve equity in the family planning market. Inspiration may be drawn from Paraguay's experience where the share of modern family planning methods used by women from the lowest income group increased by 16 percentage points (from 28% to 44%) between 2004 and 2008, because of the country's supportive policy environment removing barriers to growth of different sectors—public, private commercial, and nonprofit.^6^

India's market of family planning services and products is still dominated by the public sector and heavily subsidized by the government, which may not be healthy for the market development because oversubsidization often restricts the chance for the private sector to grow. For example, in the early 2000s, Cote d'Ivoire procured too many condoms with donor support for free distribution in urban areas,^28^ which caused difficulty for social marketing and commercial condom brands to sell their product. As a result, instead of creating new users, the free condoms were providing a free alternative to existing users who would have otherwise paid to obtain condoms.

Earlier studies family planning indicators disaggregated by socioeconomic status and residence, such as urban or rural, showed that the percentage of users of modern methods was high for higher wealth levels, and the trend has been consistent over time^20^, ^21^; however, the users of modern contraceptives in India mostly belong to higher wealth index as well. Although the market had more equity in rural areas and that did not change over the last 22 years, in the market of the urban areas, a high percentage of users of modern contraceptives still obtain the family planning services and products from subsidized sources, even though they were from relatively richer households compared to the users in rural areas.

One may argue that given the higher purchasing power of urban user, there may not be a need for continuing subsidy in urban areas; however, in India, the public health facilities in urban areas cater to a sizeable number of clients from the surrounding rural areas, who may have the need of subsidized services. In addition, high growth in number of urban poor^29^ may also be responsible for high number of clients using subsidized sources. In view of these, subsidization for the targeted groups or areas will be more useful than a “blanket” approach of removing subsidy from all urban areas.

One of the major aims of the total market approach is to make the market self‐sustainable, which is possible only if a market has enough potential to grow. At the same time, it is also critical for the users to be ready to pay for products and services. The market of family planning services and products in India shows that about 85% of the districts of India are still in the “developing” market stage—where the market is large enough but there are not enough users who are willing to pay for the products and services. Even for the urban segment of the market, where one expects higher purchasing power of the consumers, about 80% of districts are still in “developing” stage. Further, in the highest wealth segment of the market, about 70% of districts are in the “developing” stage. This reinforces the importance of targeted approach in subsidizing family planning services and products.

There is a consistent geographical variation in the stages of family planning market. The districts of the northern part of Bihar and Uttar Pradesh and districts of Northeast India are in the “early” stage. In these two areas, the market in most of the districts was in the “early” stage even among the well‐off women—for example, among women of urban areas, those belonging to the richest wealth quintile, or having high level of education. Work needs to be done in these areas to further generate demand for family planning services and products and thereby develop the market.

In fact, the Reproductive Health Primer identified key programmatic issues related to each stage of market development and suggested interventions for the total market initiatives.^1^ As the market in most of the Indian districts is at a “developing” stage, the program needs to continue building the demand as well as find ways to enhance access. The program also needs to target subsidies for low‐income consumers and matching subsidized products and services (in which both service providers and the consumers share the cost) to consumers who need them. At the same time, the intervention should focus on transitioning the users with the ability to pay from the public sector to the private sector.

This analysis of total market scenario in India has some limitations, which are inevitable because of limitations in NFHS data. In the NFHS, the respondent may not have been able to distinguish between commercial sources and partially subsidized (social marketing) sources; therefore, the percentage of unsubsidized sources may be overestimated. From the TMA point of view, the sustainability of the market will be lower than what is estimated in this analysis. The use of unsubsidized sources for pills and condoms was very high. However, about 50% of the women who obtained pills or condoms from unsubsidized sources used social marketing brands, which were generally subsidized. Some social marketing organizations distribute free samples, or often those organizations assist the public sector by distributing products free of cost at the public health facilities. Hence, some overlapping is also possible in the estimation of the market share of subsidized and unsubsidized products.

An important TMA indicator is the market volume, which indicates the number of products and services currently in the market. The NFHS data do not provide that information. The NFHS data also do not provide the information about stock outs of methods. The FPwatch survey of Bihar and Uttar Pradesh—the two high focus states of family planning program—showed less than 20% stock outs for private outlets for all kinds of nonclinical methods. Even the stock outs of pills at government‐run health subcenter or with the community health workers were around 20%.^17^

The family planning market in India showed the opportunity to the private sector, but the oversubsidization of the services challenges the growth of private sector because even the rich people avail the free or subsidized alternative of family planning services and products. The findings of this total market analysis suggest India need regional strategies to capitalize on the potentiality of the market of family planning. The geographic regions, such as, Northern parts of Bihar and Uttar Pradesh, and the Northeastern States need subsidized services to continue to generate demand. But the rest of the country needs a targeted approach with financing programs (eg, vouchers and insurance) by private financing agents to make the private sector more competitive. A healthy market of family planning services and products will benefit all sectors to grow and reach their full potential.

## FINANCIAL DISCLOSURE

This paper was prepared as part of a mentorship program under the RASTA initiative of the Evidence Project of the Population Council. The Evidence Project is made possible by the generous support of the American people through the United States Agency for International Development (USAID) under the terms of cooperative agreement no. AID‐OAA‐A‐13‐00087. The contents of this manuscript are the sole responsibility of the authors and do not necessarily reflect the views of USAID or the United States Government.

## CONFLICT OF INTEREST

None of the authors had any conflict of interest.
